# Novel smac mimetic APG-1387 elicits ovarian cancer cell killing through TNF-alpha, Ripoptosome and autophagy mediated cell death pathway

**DOI:** 10.1186/s13046-018-0703-9

**Published:** 2018-03-12

**Authors:** Bao-Xia Li, Heng-Bang Wang, Miao-Zhen Qiu, Qiu-Yun Luo, Han-Jie Yi, Xiang-Lei Yan, Wen-Tao Pan, Lu-Ping Yuan, Yu-Xin Zhang, Jian-Hua Xu, Lin Zhang, Da-Jun Yang

**Affiliations:** 1 State Key Laboratory of Oncology in South China, Collaborative Innovation Center for Cancer Medicine, Sun Yat-sen University Cancer Center, Guangzhou, 510060 China; 2 Department of Pharmacology, Fujian Provincial Key Laboratory of Natural Medicine Pharmacology, School of Pharmacy, Fujian Medical University, Fuzhou, 350108 China; 3 Ascentage Pharma Group Corp., Ltd., Taizhou, 225309 China; 4 Department of Medical Oncology, State Key Laboratory of Oncology in South China, Collaborative Innovation Center for Cancer Medicine, Sun Yat-Sen University Cancer Center, Guangzhou, 510060 China; 5 Departments of Clinical Laboratory, State Key Laboratory of Oncology in South China, Collaborative Innovation Center for Cancer Medicine, Sun Yat-Sen University Cancer Center, 651 Dongfeng Road East, Guangzhou, 510060 China

**Keywords:** APG-1387, Apoptosis, Autophagy, Ovarian cancer

## Abstract

**Background:**

Ovarian cancer is a deadly disease. Inhibitors of apoptosis proteins (IAPs) are key regulators of apoptosis and are frequently dysregulated in ovarian cancer. Overexpression of IAPs proteins has been correlated with tumorigenesis, treatment resistance and poor prognosis. Reinstalling functional cell death machinery by pharmacological inhibition of IAPs proteins may represent an attractive therapeutic strategy for treatment of ovarian cancer.

**Methods:**

CCK-8 and colony formation assay was performed to examine cytotoxic activity. Apoptosis was analyzed by fluorescence microscopy, flow cytometry and TUNEL assay. Elisa assay was used to determine TNFα protein. Caspase activity assay was used for caspase activation evaluation. Immunoprecipitation and siRNA interference were carried out for functional analysis. Western blotting analysis were carried out to test protein expression. Ovarian cancer cell xenograft nude mice model was used for in vivo efficacy evaluation.

**Results:**

APG-1387 demonstrated potent inhibitory effect on ovarian cancer cell growth and clonogenic cell survival. APG-1387 induced RIP1- and TNFα-dependent apoptotic cell death in ovarian cancer through downregulation of IAPs proteins and induction of caspase-8/FADD/RIP1 complex, which drives caspase-8 activation. NF-κB signaling pathway was activated upon APG-1387 treatment and RIP1 contributed to NF-κB activation. APG-1387 induced cytoprotective autophagy while triggering apoptosis in ovarian cancer cells and inhibition of autophagy enhanced APG-1387-induced apoptotic cell death. APG-1387 exhibited potent antitumor activity against established human ovarian cancer xenografts.

**Conclusions:**

Our results demonstrate that APG-1387 targets IAPs proteins to potently elicit apoptotic cell death in vitro and in vivo, and provide mechanistic and applicable rationale for future clinical evaluation of APG-1387 in ovarian cancer.

## Background

Ovarian cancer is the most lethal gynecological malignancy and the second most common gynecologic cancer in the world, with a high incidence of metastasis and recurrent rate [1, 2]. As one of gynecologic malignant tumors that do harm to women’s health, ovarian cancer can occur at any age. High recurrent rate and advanced stage at diagnosis are two critical challenge in the treatment of ovarian cancer [1, 3, 4]. The 5-year survival rate for ovarian cancer is only around 27% [5]. New therapeutic strategies are urgently needed in the management of ovarian cancer [6]. Despite advances intreatment strategy, many tumors are resistant to current therapeutic approaches due to defects in the apoptotic machinery of the cells [7]. For this, mechanisms of apoptosis have become promising targets for therapy [8].

Apoptosis, also called programed cell death, includes the extrinsic (type 1) and intrinsic (type 2) cell death pathways. Most of the chemotherapies kill cancer cells via the intrinsic, mitochondrial mediated cell death pathway, while some stimuli such as in the immune/inflammatory responses, TNF-alpha, FAS ligand/TRAIL, can initiate extrinsic death signals from cell surface to downstream intracellular targets. This type 1 of cell death module activates caspase-8 through its cleavage, which can then activate effector caspases 3/7, or pro-death BH3-only protein Bid. The activated or truncated Bid (tBid) translocates to mitochondria and initiates type 2 cell death process.

Many efforts have been made to explore strategies to reactivate the apoptosis in cancer cells. This has led to the development of Smac mimetics, which are designed to neutralize inhibitor of apoptosis proteins(IAPs). The IAPs are a group of anti-apoptosis proteins including cellular-IAP1 (cIAP1), cellular-IAP2(cIAP2), X-linked inhibitor of apoptosis protein(XIAP). IAP proteins are over expressed in various human malignancies and are associated with treatment resistance, disease progression and poor prognosis [9]. Smac has been found to be down-regulated in lung cancer, and decreased expression of Smac is associated with worse prognosis [10]. IAPs exert their anti-apoptotic actions through direct inhibition of initiator and effector caspases. IAPs have also been shown to ubiquitinate caspase proteins, thereby indirectly inhibit apoptosis [11–14]. Recently, several antagonists of IAPs have been developed, including APG-1387, a Smac mimetic [15]. APG-1387 and similar bivalent IAP antagonists have been shown to induce proteasomal degradation of IAPs, abrogate IAPs-mediated inhibition of caspases, and induce cell death [16, 17].

Autophagy is considered as a double-edged sword with regard to genesis, development and the treatment of tumors as it kills tumor cells but also protect tumor cells against injury [18]. To date, no studies have confrmed the role of autophagy when treated ovarian cancer with APG-1387, and the association between autophagy and apoptosis remains unclear. Therefore, the present study was to investigate the effect of APG-1387 on viability, apoptosis, clonogenic survival and autophagy in SKOV3 and OVCAR3 ovarian cancer cell lines and analyzed the association between autophagy and apoptosis. By this, we tried to reveal the potential underlying regulatory mechanism of these processes.

## Methods

### Cell cultures and reagents

Human ovarian cancer cell lines SKOV3 and OVCAR3 were purchased from the American Type Culture Collection (ATCC) provided by Sparklebio. SKOV3 and OVCAR3 cells were maintained in RPMI medium 1640 (Gibco) supplemented with 10% fetal bovine serum (Gibco, Carlsbad, CA) and 1% penicillin/streptomycin. Cells were incubated in a 5% CO2 humidified incubator at 37 °C, and collected using 0.05% trypsin EDTA following the specified incubation period. The following primary antibodies were used: P62(#8025), phospho-H_2_AX(γ-H_2_AX;#9718), caspase-8(#9746), RIP1(#3493 s), Beclin1(3738 s), ATG7(#2631S), PARP (#9546S), caspase-3(#9665 s), cIAP1(#7065 s), cIAP2(3130 s), XIAP(#14334), FADD(#2782S), phospho-NF-κBp105/p50(4806S), NF-κB2p100/p52(#4882S), TNF-α (#6945s), TNF-α neutralizing antibody (7321s), and TNFR1(#3736S) were purchased from Cell Signaling Technology Inc.; GAPDH (#sc-47724) from Santa Cruz Biotechnology (SC); LC3 (#NB100–2220) from Novus Biologicals. Z-VAD-FMK (#V116) and Necrostatin-1(#N9037) were from Sigma. IKK-16(#S2882) from Selleck. The data were collected from at least three independent experiments.

### APG-1387

The novel Smac mimetic, APG-1387 was provided by the Ascentage Pharma Group Corp. Limited (Taizhou, China). The storage concentration of APG-187 was 40 μM, stored at − 20 °C, and diluted in the corresponding culture medium just before use.

### Cell viability assay

Cells were plated in 96-well plates at a density of 5 × 10^3^ cells/ml. After 24 h, APG-1387 was added at different concentrations. Cells without APG-1387 treatment were the control group. Cells were incubated with various concentrations of drugs for 72 h. Cell viability was performed with the CCK-8 (Dojindo, Kumamoto, Japan) following the manufacturer’s instruction. The inhibition rate of cell proliferation was calculated for each well as (A450_control cells_ – A450 _treated cells_)/A450_control cells_ × 100%. Experiments were performed in triplicate. Cell viability was expressed as mean ± SD of absorbance from treated cells vs. control cells in triplicate.

### Apoptosis analysis *by Fluorescence microscopy*

APG-1387-induced apoptosis was assessed by Hoechst33258 staining. Treated with 10 nM APG-1387 for 24 h, the cells were harvested and smeared on slides. The slides were air-dried, fixed in methanol-acetone (3/1, *v*/v), and stained with Hoechst33258 (5 μg/mL) at 37 °C for 20 min. Nuclear morphology was examined under fluorescence microscopy (DFC480; LeicaMicrosystems, Wetzlar, Germany) to identify cells undergoing apoptosis.

### Apoptosis analysis by flow cytometry

Apoptosis was detected by an AnnexinV-propidium iodide (PI) apoptosis detection kit (4A Biotech Company Limited, FXP018–100; Beijing, China) as described previously [19]. To determine the apoptotic rate, the cells were placed in 6-well plates and treated with APG-1387 (0, 10 nM, 30 nM) at a density of 2 × 10^5^ cells/well for 24 h, and then collected. AnnexinV-FITC/PI (Becton Dickinson, USA) staining was performed, following the manufacturer’s protocol. The apoptotic rate was determined using Celluest software (FCM, Becton Dickinson, USA).

### Colony formation assay

Colony formation assay was used to evaluate the effects of APG-1387 on the proliferation of ovarian cancer. The cells were cultured in 6-well microplates (300 cells/ well), Then cells were treated with indicated concentrations of APG-1387 in microplates for 7 days. Cells were stained with crystal violet (Sigma, St. Louis, Mo, USA) for 20 min. Images of the colonies were obtained using a digital camera (Canon, EOS350D, Tokyo, Japan).

### ELISA

ELISA Kit (Cusabio Biotech Co., Ltd., Wuhan, China) were used for TNF-alpha detection, according to manufacturer’s instructions. The absorbance was measured in an ELISA reader at 450 nm. The concentrations of cytokines were calculated according to the standard curve using each of the recombinant cytokines in the ELISA kits.

### Immunoprecipitation

Cells were cultured in 10 cm plates, treated as indicated, washed twice with PBS, and harvested with 1 ml of the following lysis buffer: 20 mM TrisHCl (pH 7.5), 150 mM NaCl, 10% glycerol, 1% TritonX-100, 2 mM EDTA, with complete, EDTA-free protease inhibitor (Roche) and phosphatase inhibitors (Sigma). Cells were left on ice for 30 min and centrifuged at 12,000 rpm for 20 min. Three milligrams of protein lysate were used for immunoprecipitation using 2 mg of the antibodies overnight at 4 °C. The following day, 25 μl of protein A/G ultralink resin (Thermo Scientific) was added for 2 h at 4 °C. The IPs were washed three times with lysis buffer, then sample buffer was added and the beads were boiled for 5 min at 95 °C. The samples were then analyzed by SDS-PAGE followed by immunoblotting with the indicated antibodies.

### Caspase activity assay

After the applied APG-1387 treatment, cytosolic proteins (30 mg pertreatment) were incubated with the caspase assay buffer, with corresponding caspase substrate (AcIETD-AFC for caspase-8 and Ac-DEVD-AFC for caspase-3). Caspase activity was performed with the caspase-8 Activity Assay Kit (Beyotime Biotechnology, Shanghai, China) and caspase-3 Activity Assay Kit (Beyotime Biotechnology, China) following the manufacturer’s specifications. The plates were analyzed on an automated microplate spectrophotometer (Thermo Molecular De-vices Co., Union City, USA) at 405 nm.

### siRNA and GFP-LC3 interference

The target sequence for Beclin1-specific siRNAs were 5′-UGGAAUGGAAUGAGAUUAAT-3′and 5′-UGGAAUGGAAUGAGAUUAATT-3′, 5′-GCTGCCGTTATACTGTTCT-3′ another target sequences ATG7-specific siRNAs were 5′-GCCGUGGAAUUGAUGGUAUTT-3′, 5′-GAAGCUCCCAAGGACAUUATT-3′ and 5′-GGAUCCUGGACUCUCUAAATT-3′, for all of which and the control siRNA (no silencing) were synthesized by GenChem Co. (Shanghai, China). The plasmid GFP-LC3 was kindly provided by Beth Levine. Transfection was performed, following the manufacturer’s protocol.

### Immunocytochemistry

Immunofluorescence staining was conducted using a procedure similar to that described previously [20]. The cells were plated on sterile coverslips and treated with 0 nM (APG-1387 control group) and 3 nM APG-1387 for 24 h, and fixed with 4% paraformaldehyde (PFA) for 10 min at 37 °C. After fixation, a permeabilization step was conducted with 0.25% Triton-X 100 for 10 min at 4 °C, and the cells were subsequently incubated in blocking solution containing 4% bovine serum albumin (BSA) for 1 h at 37 °C. The nucleus was stained with DAPI (1 mg/ml) for 5 min at room temperature. Fluorescence images were then captured by a confocal laser scanning microscope (LSM 700) (Carl Zeiss, Oberkochen, Germany).

### Western blot assays

Cells were treated with various concentrations of drugs for 24 h, harvested in lysis buffer and tumors were harvested in RIPA buffer. After incubating on ice for 30 min, cells were centrifuged at 12,000 g for 15 min at 4 °C, and supernatant was collected. Samples were then analyzed by western blot. Proteins were visualized by incubation with SuperSignal west pico reagents (NCI5079, Thermo), followed by exposure to radiograph film.

### Nude mouse xenograft studies

Four-week-old BALB/c (athymic) nude mice were purchased from the Shanghai’SIPPR-BK laboratory animal Co., Ltd.. The animal study protocol was approved by institutional animal care and use committee (IACUC) at Ascentage. All animal experiments were performed at Ascentage. A total of 5 × 10^6^ skov3 cells were subcutaneously injected into the right flank of nude mice. When tumor volume reached 100–150 mm^3^ on average, the mice were randomized into 6 treatment groups. APG-1387 or vehicle control was administered intravenously in a total volume of 200 μL at the indicated dosing schedules. Body weights and tumor volumes (V) were measured every 2 days. Tumor volumes were calculated according to the formula: V = (lenght × width^2^)/2.

#### Immunohistochemistry (IHC) staining

Xenografts were fixed, and embedded in paraffin; Tissue sections (4-mm) were blocked with 0.5% BSA. Cell apoptosis was detected by terminal deoxynucleotidyl transferased UTP nick end labeling (TUNEL) In Situ Cell Death Detection Kit (Roche), following the manufacturer’s specifications. We counted the number of positive cells for TUNEL in all areas of the section under a light microscope at × 20 magnification and calculated the number of TUNEL-positive cells per field.

### Statistical analysis

All assays were performed in triplicate. Data are expressed as the mean ± SD. Statistical analyses were performed using an analysis of variance with SPSS 13.0 software. Statistical significance was set at two-sided *P* < 0.05.

## Results

### Growth inhibition effects of APG-1387 on ovarian cancer

**Fig. 1:**
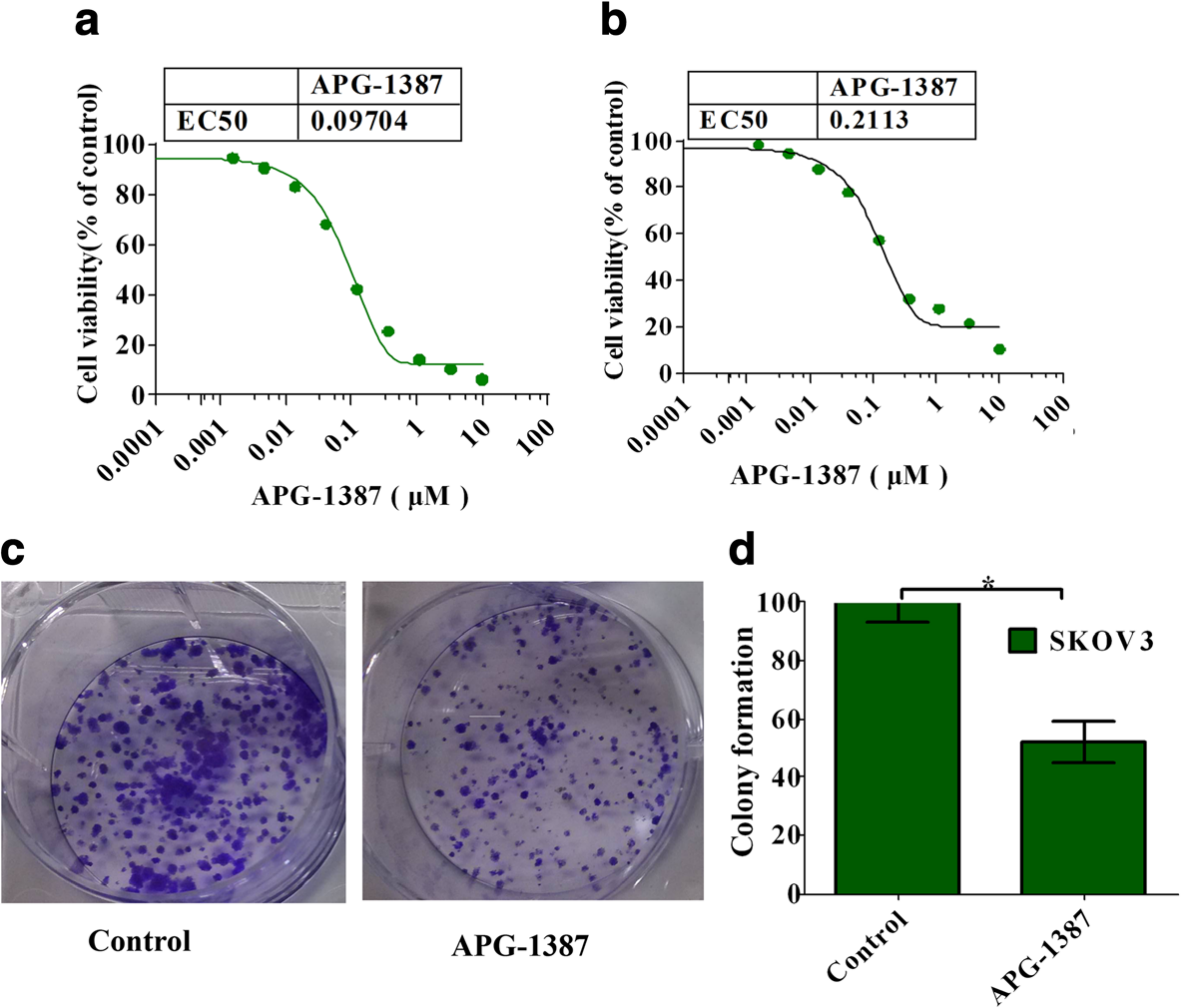
Effects of APG-1387 on cell viability in ovarian cancer. **a**, **b** APG-1387 inhibited the proliferation of SKOV3 and OVCAR3 cell lines. They were treated with the indicated concentrations of APG-1387 for 72 h and cell viability was determined by the CCK-8 assay. **c** Colony-forming test results. SKOV3 cells were incubated with APG-1387 (0, 3 nM) for 7 days. **d** Statistical analysis of the percentage of clone numbers. Columns, mean (*n* = 3); bars, SD. *P < 0.05 vs. untreated group

In order to investigate the antitumor activity of APG-1387, we evaluated the effect of APG-1387 on cell growth of human ovarian cancer cell lines SKOV3 and OVCAR3 after 72 h of treatment, as shown in Fig. 1a, b. The inhibitory effect of APG-1387 on the growth of SKOV3 and OVCAR3 cells were assessed by the CCK-8 assay. The various concentrations of APG-1387 inhibited the viability of SKOV3 and OVCAR3 cells in both a dose-and time-dependent manner, and the EC_50_ value were 0.097 μM and 0.2113 μM respectively. In addition, colony formation assay showed that APG-1387 strongly decreased survival of ovarian cancer cells (Fig. 1c, d). These results indicate that APG-1387 strongly inhibited the growth and survival of tumor cells in a dose- and time-dependent manner.

### APG-1387 induces apoptosis and activates caspase-3/PARP in ovarian cancer

**Fig. 2:**
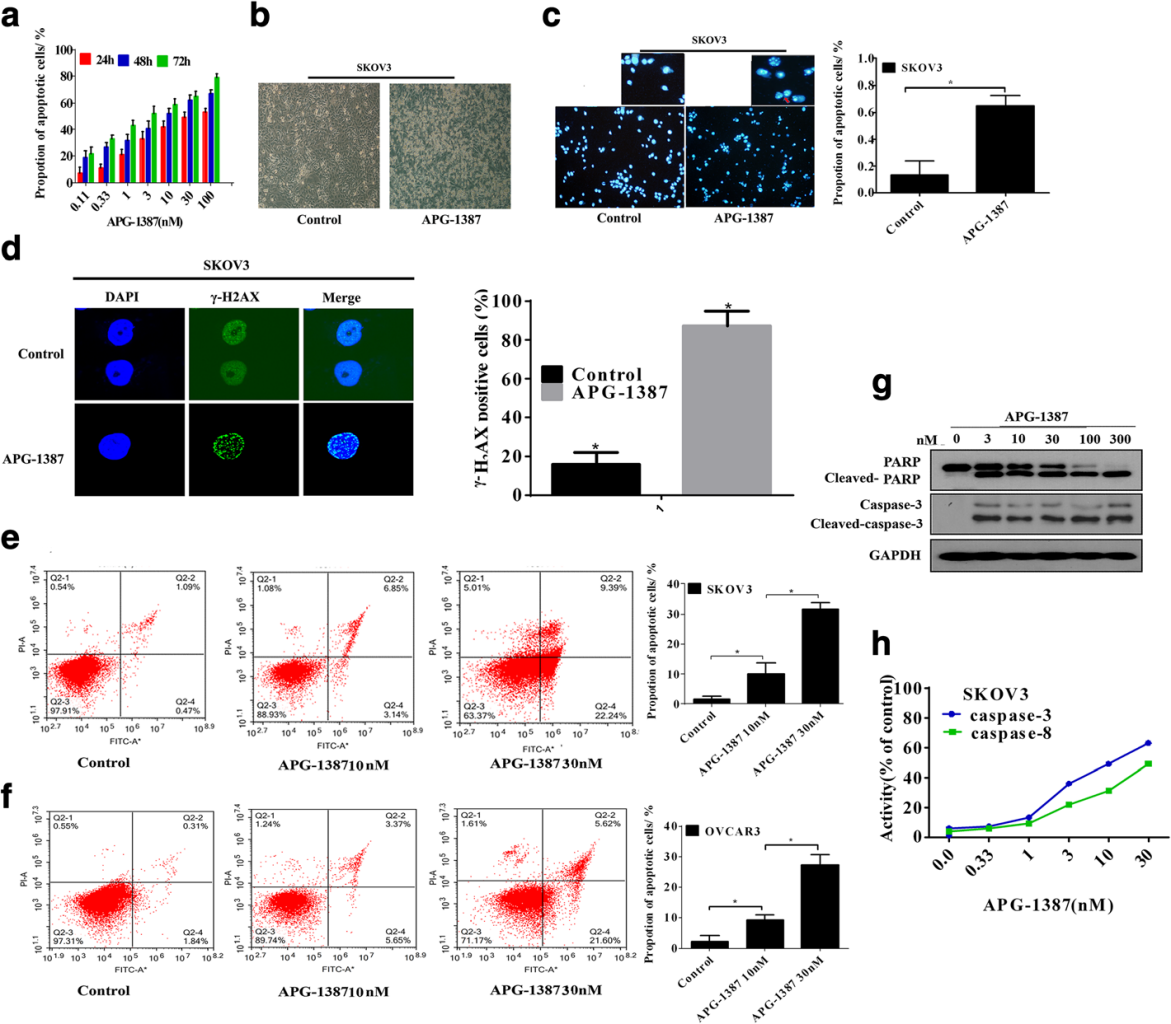
Effects of APG-1387 on apoptosis in ovarian cancer. **a**. APG-1387 inhibited the proliferation of SKOV3 cell line. They were treated with the indicated concentrations of APG-1387 for 24, 48, 72 h. Cell viability was determined by the CCK-8 assay. **b** Morphology of SKOV3 cells exposed to APG-1387(0, 10 nM) photographed under a fluorescence microscope (original magnification× 10). **c** APG-1387-induced apoptosis in SKOV3 cells was assessed by Hoechst33258 staining. Morphology of SKOV3 cells exposed to APG-1387 at different concentrations photographed under a fluorescence microscope (original magnification × 10). Condensated and fragmented nuclears were the mean ± SEM of 5 randomized areas. *P* < 0.01. **d** SKOV3 cells were treated with 10 nM APG-1387 for the indicated times. The cells were stained for phosphorylated H_2_AX and DAPI, then were analyzed by fluorescence microscopy (original magnification × 200). γ-H_2_AX positive spots were the mean ± SEM of 5 randomized areas. *P* < 0.01. **e**, **f** SKOV3 and OVCAR3 cells were exposed to various concentrations of APG-1387 (0, 10, 30 nM) for 24 h followed cell apoptosis analysis by flow cytometry. **g** Western blot analysis of caspase-3/PARP SKOV3 cells were treated withAPG-1387 (0, 3, 10, 30, 100, 300 nM) for 24 h. The data shown are representative of three different experiments. **h** SKOV3 cells were stimulated with APG-1387 for indicated periods of concentrations, caspase activation were tested by caspase activity assay

In this study, we evaluated the effects of APG-1387 on cell apoptosis of human ovarian cancer cell line SKOV3 after 24, 48, 72 h of treatment, as shown in Fig. 2a. Cell morphology examination revealed that the majority of the APG-1387-treated cells presented the characteristics of shrinkage, irregularity compared with mock-treated cells (Fig. 2b), indicating cell damage induced by the APG-1387 treatment. Apoptotic nuclear morphology was observed after Hoechst33258 staining using fluorescence microscopy. After treatment with 10 nM APG-1387 for 24 h, SKOV3 cells began to exhibit apoptotic characteristics, such as nuclear condensation, and fragmentation. In the control group, the cells were regular in morphology and grew fully in patches and were confluent, rarely sloughing off (Fig. 2c). Although DNA damage is a generic term for many different DNA modifications that have the ability to activate apoptosis, APG-1387 treatment caused the formation of significant amounts of double-stranded DNA breaks (DSBs), as indicated by the phosphorylation of H_2_AX (Fig. 2d). Apoptosis detection by AnnexinV and PI staining showed that when APG-1387-treated SKOV3 and OVCAR3 cells had increased apoptosis rates at concentration of 10 nM and 30 nM than the control group (*P* < 0.05). As shown in Fig. 2e and f, the proportion of apoptotic SKOV3 cells increased from 1.56% to 31.63% and the proportion of apoptotic OVCAR3 cells increased from 2.15% to 27.22% after treatment with different concentrations of APG-1387, suggesting that APG-1387 induced cell apoptosis in a dose-dependent manner in SKOV3 and OVCAR3 cells. In this study, we investigated caspases-3/PARP in APG-1387-induced apoptosis. As showed in Fig. 2g, APG-1387 activated caspases-3/PARP in a dose-dependent manner, Thus, APG-1387 induced ovarian cancer apoptosis. Next, the caspase-3/8 activity was also examined. Figure 2h demonstrated that APG-1387 dose-dependently increased the activity of caspase-3/8 in SKOV3 cells.

### APG-1387-induced cell death is caspase-dependent

**Fig. 3:**
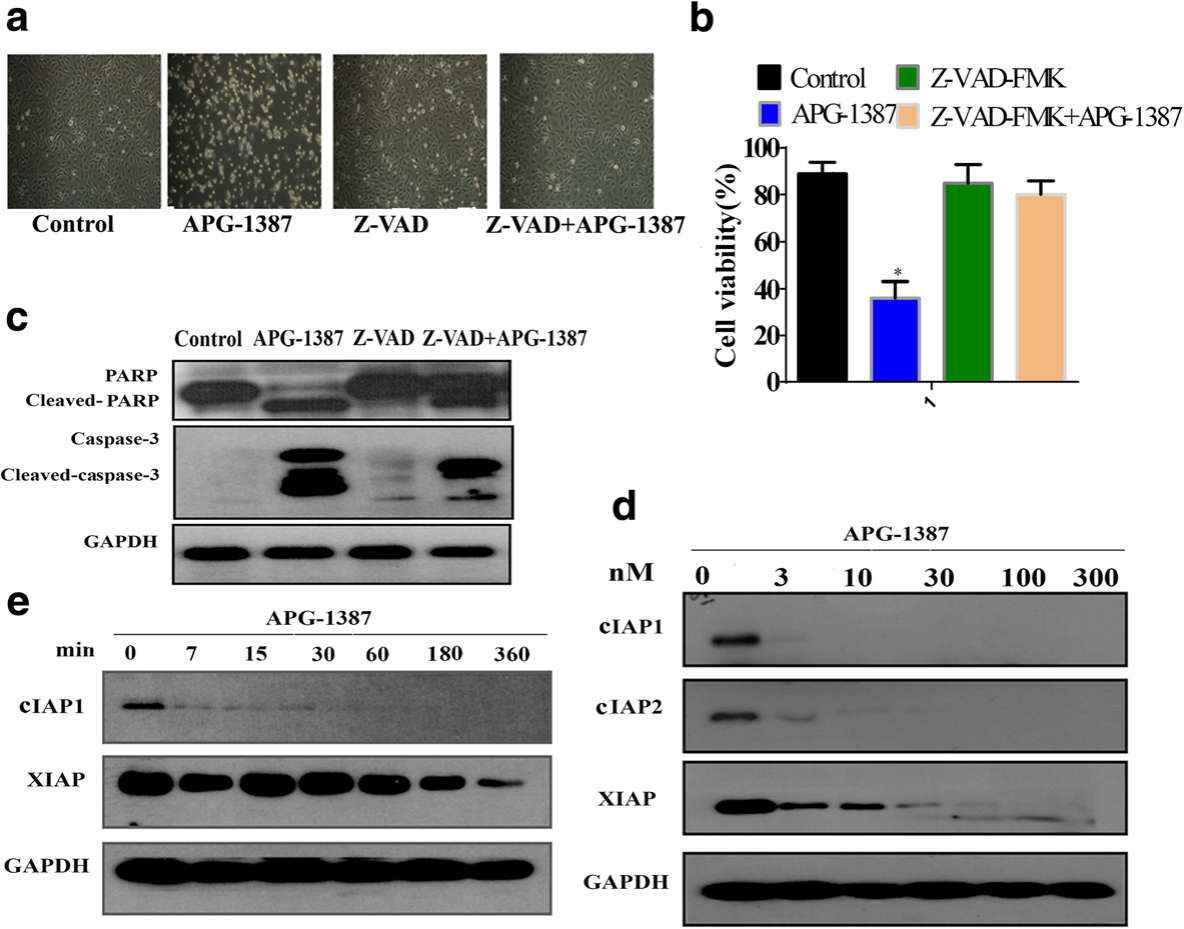
APG-1387-induced apoptosis in caspase dependent manner. **a** Cells with or without addition of Z-VAD-FMK. Morphology of cells exposed to different treatment groups photographed under a fluorescence microscope (original magnification × 10). **b** APG-1387 was coadministered with or without addition of caspase inhibitor (Z-VAD-FMK). Cell viability was determined by the CCK-8 assay. **c** Western blot analysis of the effect of APG-1387 with or without addition of Z-VAD-FMK on caspase-3/PARP expression level in SKOV3 cells. **d** Western blot analysis of the expression levels of IAPs at different concentrations of APG-1387 in SKOV3 cells. **e** Cells were treated with different time points, and the effect of APG-1387 on IAP family members expression level was determined by western blot. Data represent one of three experiments yielding similar results

To determine whether the cell death induced by APG-1387 was caspase-dependent, APG-1387 was coadministered to SKOV3 cells with or without Z-VAD-FMK, which is a pan caspase inhibitor. In SKOV3 cells, the growth inhibition of cell morphology and cell viability were partially blocked by addition of Z-VAD-FMK (Fig. 3a, b), and at the same time we found that the activation of caspase-3 and PARP were also partially blocked by addition of Z-VAD-FMK (Fig. 3c). Meanwhile, we examined the protein levels of cIAP1, cIAP2 and XIAP in SKOV3 cells by western blot in APG-1387-induced apoptosis. A concentration and time course of SKOV3 cells’ response to APG-1387 treatment showed that the degradation of cIAP1, cIAP2 and XIAP was in a dose-and time-dependent manner (Fig. 3d, e).

### APG-1387 is RIP1-dependent in ovarian cancer induced apoptosis

**Fig. 4:**
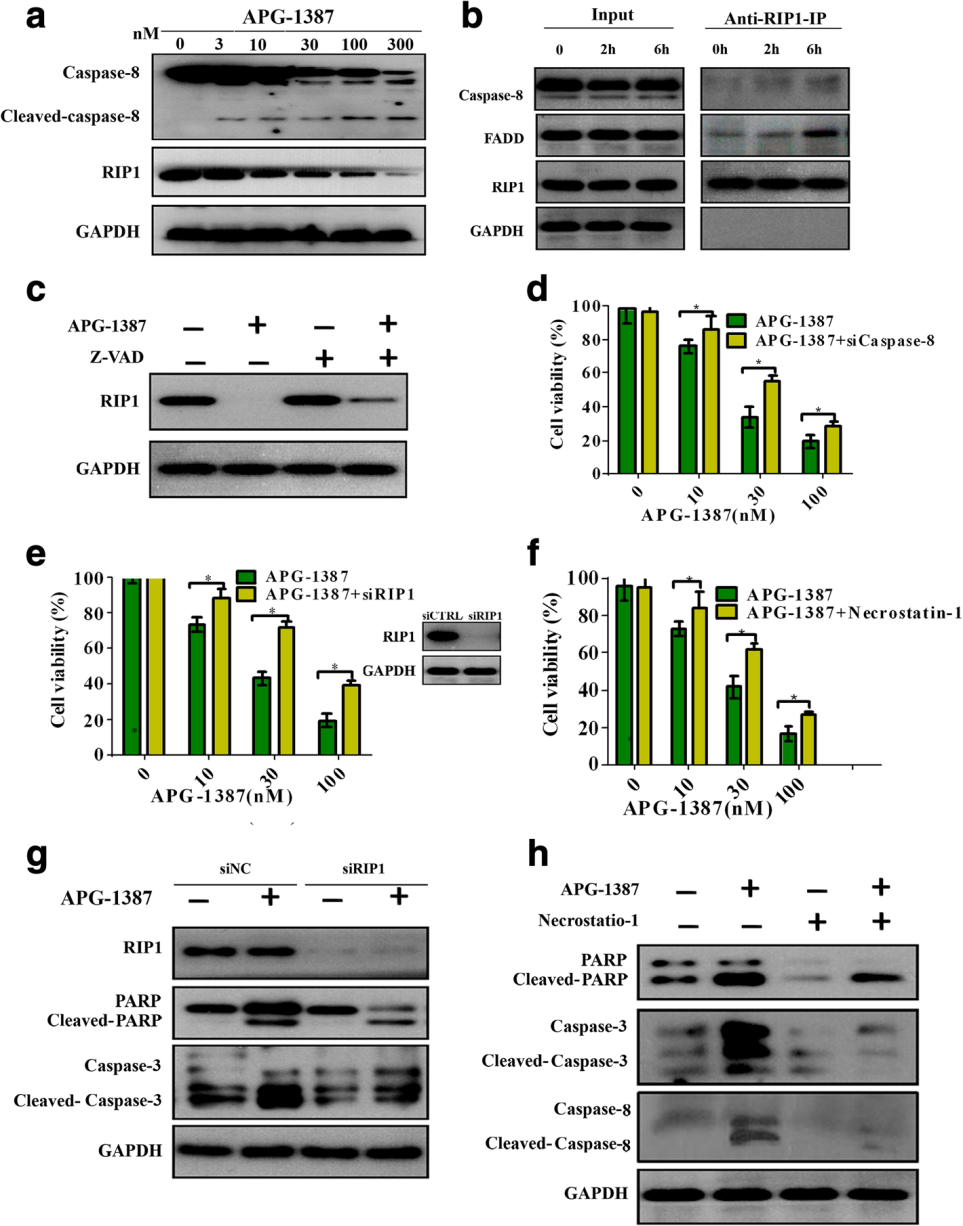
APG-1387 is RIP1-dependent in ovarian cancer induced apoptosis. **a** Western blot analysis of the expression levels of caspases-8 and RIP1 at different concentrations of APG-1387 in cells. **b** Cells were treated with APG-1387 (10 nM) for the indicated times. The indicated proteins were detected by western blot after co-Immunoprecipitation with an antibody for RIP1, GADPH was detected as a control for specificity of co-Immunoprecipitation. **c** Western blot analysis of the effect of APG-1387 with or without addition of Z-VAD-FMK on RIP1 expression level in the SKOV3 cells. **d** Cells were transfected with caspases-8 siRNAs, cell viability was determined by the CCK-8 assay. **e** Cells were transfected with RIP1 siRNAs, cell viability was determined by the CCK-8 assay. **f** APG-1387 was coadministered with or without Necrostatin-1, cell viability was determined by the CCK-8 assay. **g** Cells were transfected with RIP1 siRNAs. Western blot analysis of the expression levels of caspases-3 and PARP. **h** The protein levels of cleaved-caspases-3/8 and PARP in cells by western blot after APG-1387 was coadministered with or without Necrostatin-1

We examined the protein levels of caspase-8/RIP1 by western blot. APG-1387 triggered the activation of caspase-8 and downregulated the protein level of RIP1, as shown in Fig. 4a. We explored the complex consisting of RIP1, FADD and caspase-8. Co-immunoprecipitation was employed to characterize the binding partners of FADD. After treatment with APG-1387, RIP1-containing complexes were analyzed by western blot, revealing the expected binding between RIP1 and caspase-8 (Fig. 4b). Under some conditions, cells undergo apoptosis and necrosis simultaneously [19], APG-1387 was coadministered to SKOV3 cells with or without Z-VAD-FMK. In SKOV3 cells, we found that the expression of RIP1 were also partially reversed by addition of Z-VAD-FMK (Fig. 4c). Next, cells were transfected with caspases-8 siRNAs, the cell viability was partially reversed by transfected with caspases-8 siRNAs, as shown in Fig. 4d. To determine whether the cell death induced by APG-1387 was RIP1-dependent in ovarian cancer or not, cells were transfected with RIP1 siRNAs. The cell viability was partially reversed by transfected with RIP1 siRNAs, as shown in Fig. 4e. Next, APG-1387 was coadministered with or without Necrostatin-1, which is a specific RIP1 inhibitor. Cell viability was partially reversed, as shown in Fig. 4f. We also examined the protein levels of caspases-3/PARP by western blot, after cells were transfected with RIP1 siRNAs (Fig. 4g) or when APG-1387 was coadministered with Necrostatin-1 compared with APG-1387 alone (Fig. 4h). The protein levels of cleaved-caspases-3/8 and cleaved-PARP was also partially reversed, when cells were transfected with RIP1 siRNAs or APG-1387 was coadministered with Necrostatin-1compared with the use of APG-1387 alone.

### APG-1387 induces apoptotic cell death through engagement of TNFR1 by TNF-alpha signaling pathway

**Fig. 5:**
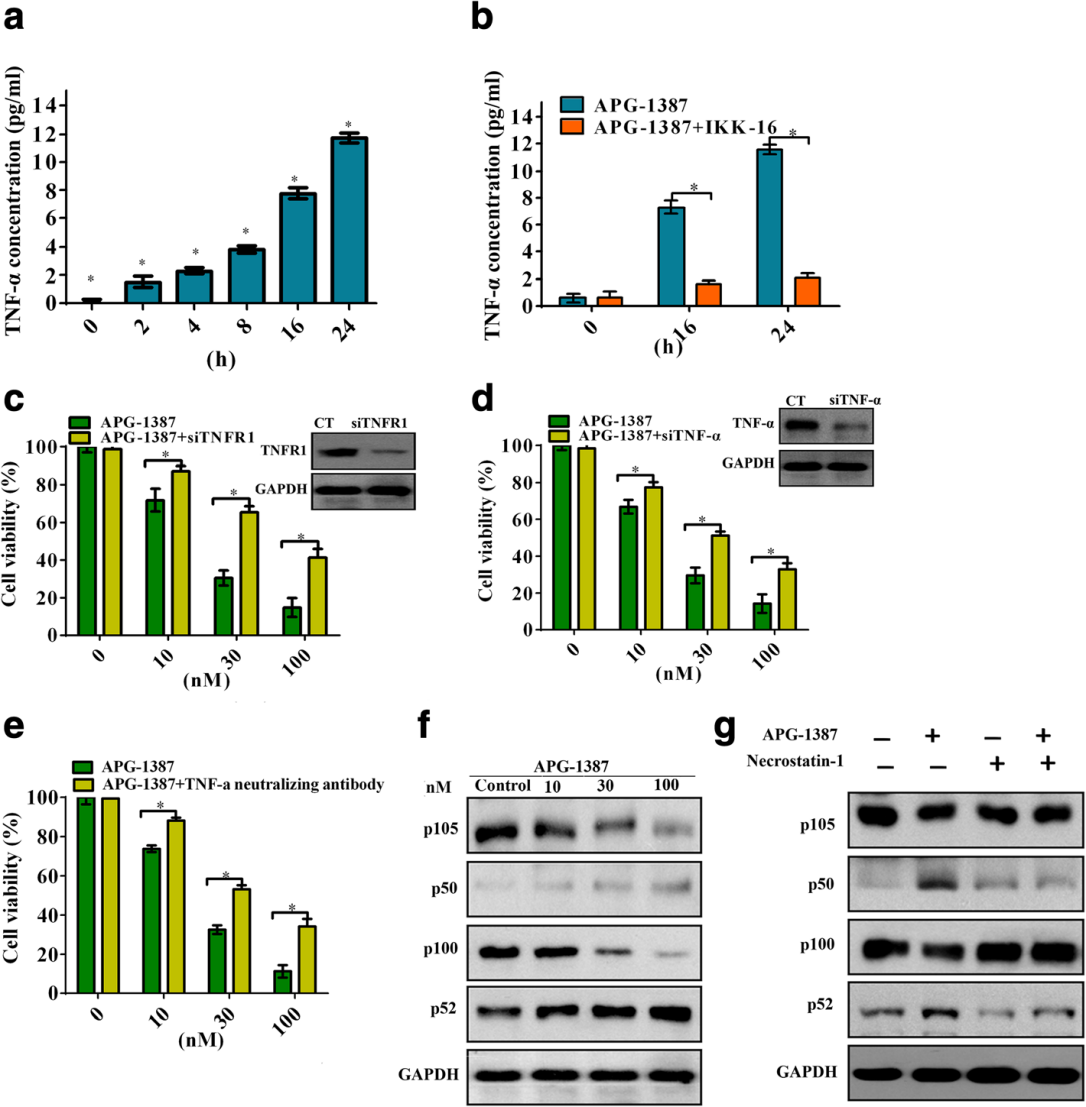
APG-1387 induces cell death partly through TNF-alpha signaling pathway. **a** ELISA analysis of the secretion of TNF-alpha after different time points of APG-1387 on SKOV3 cells streatment. **b** Cells were treated with different time points of APG-1387, and the secretion of TNF-alpha were analyzed by ELISA. **c** Cells were transfected with TNFR1 siRNAs, cell viability was determined by the CCK-8 assay. **d** Cells were transfected with TNF-alpha siRNAs, Cell viability was determined by the CCK-8 assay. **e** APG-1387 was coadministered with or without TNF-alpha neutralizing antibody, cell viability was determined by the CCK-8 assay. **f** Cells were treated with different concentrations of APG-1387 on cells after 24 h treatment and the expression levels of NF-κB1/p50 and NF-κB2/p52 were determined by western blot. **g** The protein levels of NF-κB1/p50 and NF-κB2/p52 in cells by western blot after APG-1387 was coadministered with or without Necrostatin-1

We examined the secretion of TNF-alpha after SKOV3 cells were treated with APG-1387, and found that the secretion of TNF-alpha was in time-dependent manner (Fig. 5a). Additionally, IKK-16, which is a selective IκB kinase (IKK) inhibitor, resulted in a dramatic decrease in the secretion of TNF-alpha (Fig. 5b). Transfection of cells with siTNFR1, cell viability was increased significantly (Fig. 5c). Transfection of SKOV3 cells with siTNF-alpha, cell viability was also increased significantly (Fig. 5d). Next, APG-1387 was coadministered with or without TNF-alpha neutralizing antibody, cell viability was also partially reversed, as shown in Fig. 5e. These results demonstrate that TNFα signaling is required for APG-1387-induced apoptotic cell death. Next, we have investigated the expression of NF-κB1/p50 and NF-κB2/p52 by western blot after cells were incubated with various concentrations of APG-1387. Our results showed that NF-κB1/p50 and NF-κB2/p52 were activated, as shown in Fig. 5f. Mechanistic investigations revealed that the proliferative effects of RIP1 over expression were mediated by NF-kB activation [20]. In our study, we next investigated the role of RIP1 in the activation of NF-κB after cells was coadministered with or without Necrostatin-1. We found that RIP-1 was required for NF-kB activation, as shown in Fig. 5g.

### APG-1387 induces autophagy in ovarian cancer

**Fig. 6:**
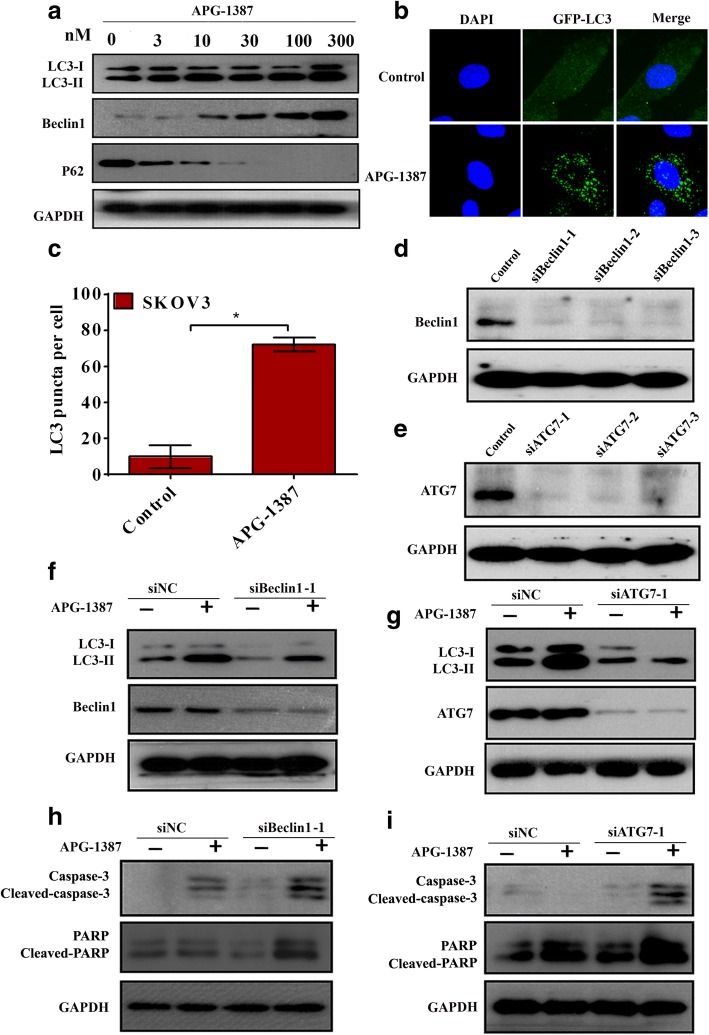
APG-1387 induces autophagy in ovarian cancer cells. **a** The expression of LC3, Beclin1 and P62 was measured by western blot. Cells were treated with APG-1387(0, 3, 10, 30, 100, 300nM) for 24 h. **b** Cells were transfected with GFP-LC3 plasmids, and then maintained in media with or without 3 nM APG-1387 for 24 h. The cells were then stained with DAPI and analyzed by fluorescence microscopy. **c** Statistical analysis of the percentage of LC3 puncta per cell. Columns, mean (*n*=3); bars, SD. *P<0.01 vs. untreated group. LC3 puncta per cell were quantified. **d** Cells were transfected with Beclin1 siRNAs. Western blot was used to detect the expression of Beclin1. **e** Cells were transfected with ATG7 siRNAs. Western blot was used to detect the expression of ATG7. **f** Cells were transfected with Beclin1 siRNAs. After 24 h treatment with or without 3 nM APG-1387, western blot analysis was performed for indicated proteins. **g** Cells were transfected with ATG7 siRNAs. After 24 h treatment with or without 3 nM APG-1387, western blot analysis was performed for indicated proteins. **h** Western blot analysis was performed for indicated proteins in cells transfected with siBeclin-1 and treated with 10 nM APG-1387. **i** Western blot analysis was performed for indicated proteins in cells transfected with siATG7-1 and treated with 10 nM APG-1387

Autophagy was measured by light microscopic quantitation of cells transfected with GFP-LC3 as described or by western blot analysis of the levels of LC3, Beclin1 and P62 [21, 22]. To investigate APG-1387-induced autophagy, we measured the expression levels of LC3 and Beclin1, as shown in Fig. 6a. To further explain these results, the level of P62, a substrate that is degraded during autophagy [23], was also assessed in SKOV3 cells. The intensity of the LC3-II band increased and the conversion from LC3-I into LC3-II was evident after treatment with APG-1387, indicating the activation of autophagy by APG-1387. To further confirm these results, we observed LC3 punctate formation under a confocal microscope. LC3 is an autophagosome membrane marker, as demonstrated by accumulation of punctate LC3 in the cytoplasm of cells (shown in Fig. 6b, c), suggesting that APG-1387 induced autophagy in SKOV3 cells. To further confirm our hypothesis, siRNAs were designed to reduce Beclin1 and ATG7 protein levels and these siRNAs completely abolished the expression of Beclin1and ATG7 (shown in Fig. 6d, e). Beclin1 and ATG7 protein knockdown dramatically counteracted the autophagy induced by APG-1387 treatment (Fig. 6f, g), confirming that APG-1387-induced autophagy. Next, we investigated the expression of cleaved-caspase-3 and cleaved-PARP When APG-1387 was coadministered with Beclin1 siRNAs and ATG7 siRNAs. When APG-1387 was coadministered with Beclin1 siRNAs and ATG7 siRNAs, increasing cleavage of caspase-3 and PARP was found, as shown in Fig. 6h, i, suggesting a cytoprotective role of autophagy triggered by APG-1387 in SKOV-3 cells.

### Inhibition of autophagy sensitizes ovarian cancer cells to A PG-1387-induced apoptosis

**Fig. 7:**
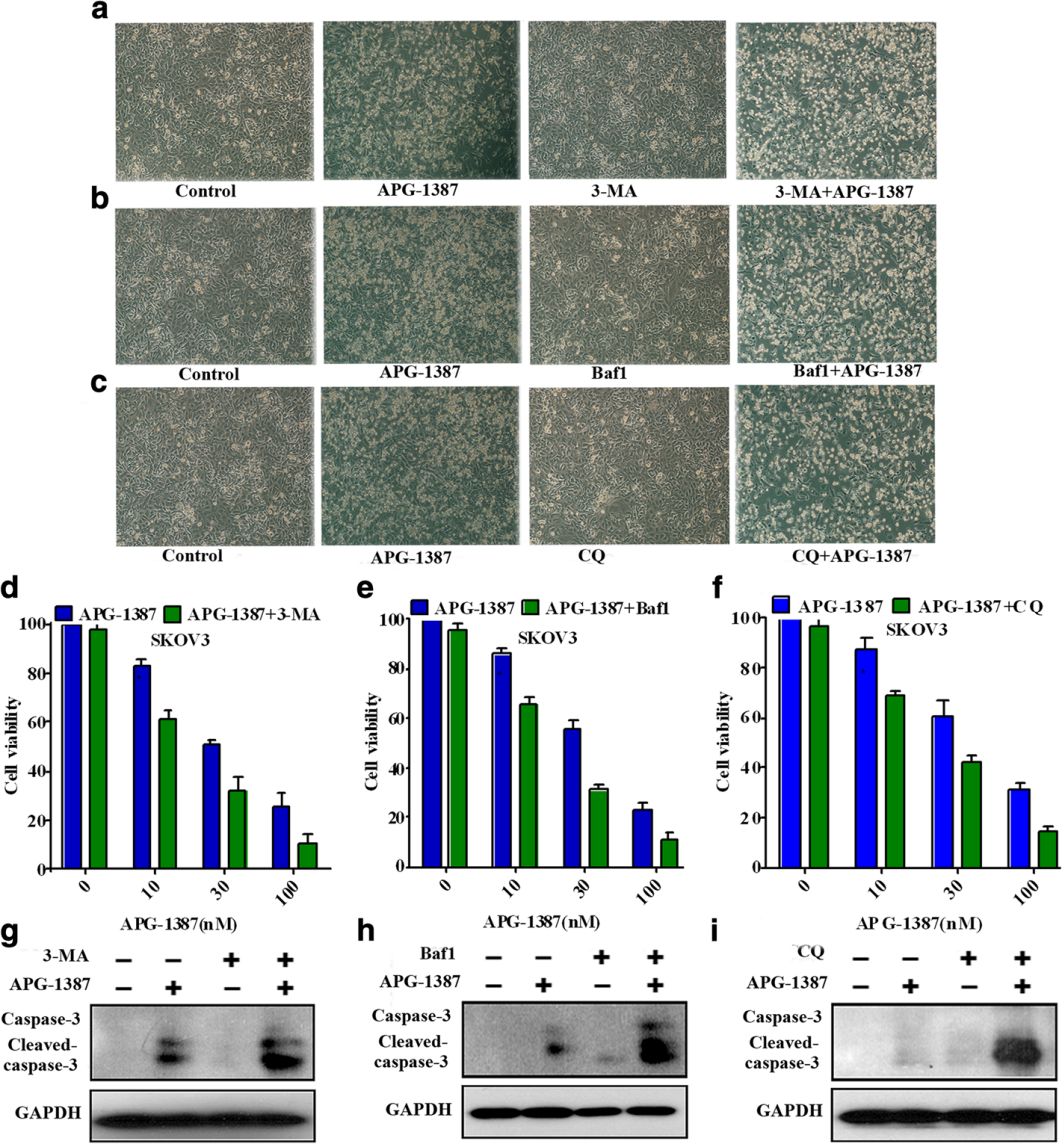
APG-1387-induced autophagy promotes cell survival in SKOV3 cells. **a, b, c** The cell death was clearly evident in all cell lines by phase-contrast microscopy, Cells were incubated for 2 h in the presence or absence of 3-MA (20 mM), Baf A1 (100 nM) and CQ (10 μM), then, APG-1387 combined with or without 3-MA (20 mM), Baf A1 (100 nM) and CQ (10 μM) for 24 h. **d, e, f** Cells were incubated for 2 h in the presence or absence of 3-MA, Baf A1 and CQ, then, Cell viability were tested after APG-1387 combined with or without 3-MA, Baf A1 and CQ treatment 24 h was determined by the CCK-8 assay. **g**, **h**, **i** The expression of caspase-3 was measured by western blot. Cells were incubated for 2 h in the presence or absence of 3-MA, Baf A1 and CQ, then, APG-1387 combined with or without 3-MA, Baf A1 and CQ for 24 h. The data shown are representative of three different experiments

In our study, we next investigated the role of autophagy in APG-1387-induced cytotoxicity with different autophagy inhibitors: 3-methyladenine (3-MA), BafilomycinA1 (Baf-A1), and CQ. These inhibitors block autophagy at different steps along the autophagic pathway. Although cytotoxicity caused by APG-1387 alone was detected, a significant suppression of APG-1387 combined with 3-MA, BafA1, CQ-induced cell death was partially observed (Fig. 7a–f). Inhibition of autophagy by 3-MA, BafA1, CQ promoted cells against APG-1387-inducing death, which was detected by cell viability assay. When APG-1387 was coadministered with 3-MA, BafA1, CQ, cell viability decreased, as shown in Fig. 7d–f. These observations were further supported by western blot. We investigated the expression levels of caspase-3 after APG-1387 combined with or without 3-MA, BafA1 and CQ. When APG-1387 was coadministered with 3-MA, BafA1, CQ, increasing cleavage of caspase-3 was found, as shown in Fig. 7g–i. It indicated that autophagy played a role in promoting tumor cell growth and survival.

### APG-1387 inhibits tumor growth as a single agent in xenografted ovarian models

**Fig. 8:**
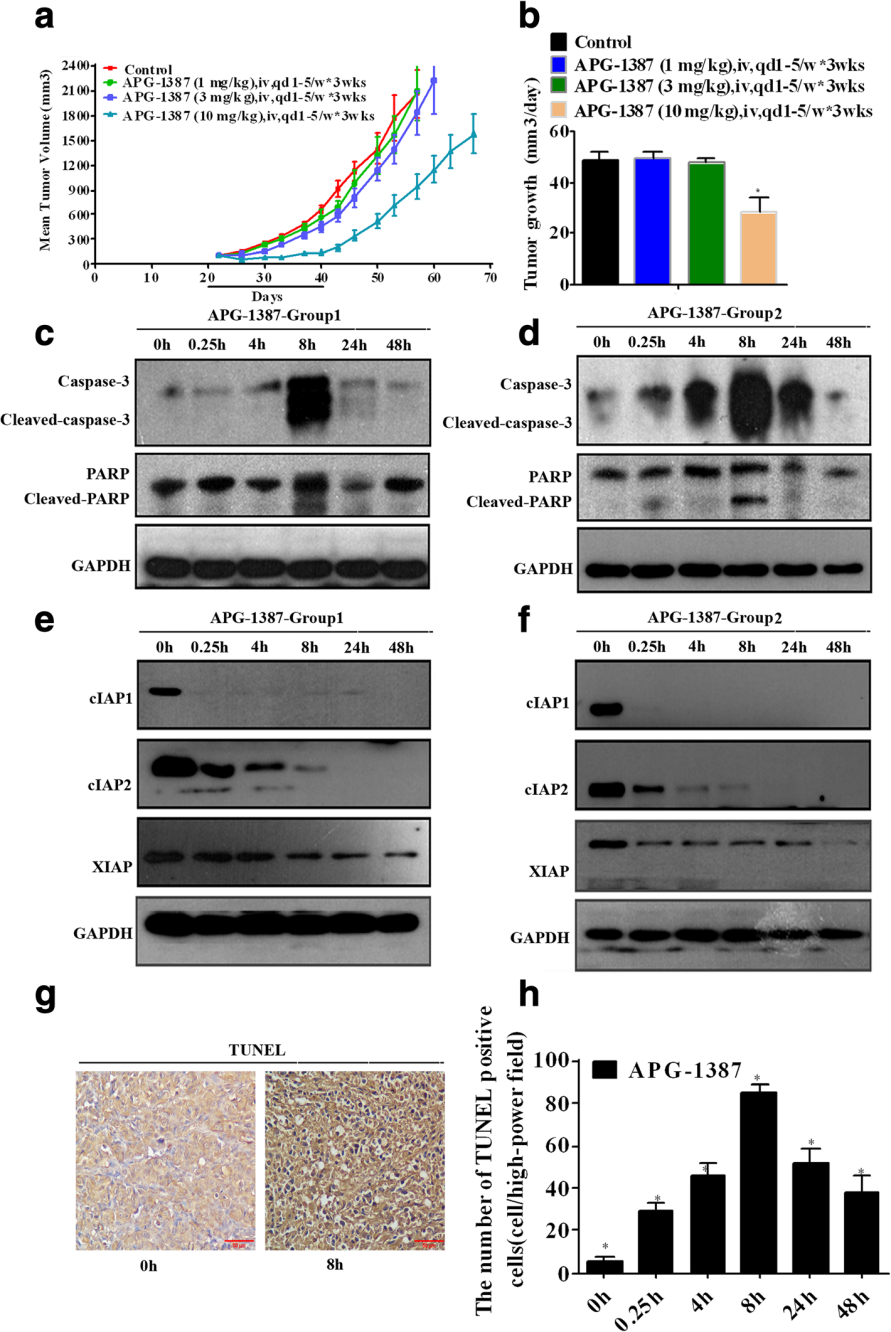
APG-1387 inhibits tumor growth in xenografted ovarian models. **a, b** Changes in tumor volume and tumor growth between APG-1387 (1, 3, 10 mg/kg) group and vehicle group of SKOV3 tumor-bearing mice. Data are shown as mean tumor volume ± SD (eight mice/group). **c, e, d, f** Mice bearing SKOV3 xenograft tumor were treated with a single intravenous dose of APG-1387 and tumors were collected at the indicated time points for western blot analysis (Group 1: **c, e** and Group 1: **d, f**) or TUNEL assay (**g, h**). For western blot analysis, tumor lysates were assigned into two groups (Group 1: **c, e** and Group 1: **d, f**) and analyzed for the indicated proteins. **g, h** IHC staining assay (testing TUNEL-positive cells). All values were expressed as mean ± SD. *p < 0.05 vs. group of “control”. Bar ¼ 100 mm

We tested the potential anti-cancer activity of APG-1387 in vivo. The SKOV3 nude mice xenograft model was utilized. As shown in Fig. 8a, APG-1387 at 1, 3 and 10 mg/kg significantly inhibited SKOV3 tumor growth in mice. Treatment with APG-1387 also resulted in reduced tumor weight in SKOV3 model (Fig. 8b). Animals treated with APG-1387 showed no signs of associated toxicity, indicating that APG-1387 at doses up to 10 mg/kg was well-tolerated. To explore the mechanism, nude mice bearing SKOV3 xenograft tumor were treated with a single intravenous dose of APG-1387 at 10 mg/kg and tumors were collected at the indicated time points for western blot analysis and TUNEL assay. Consistent with in vitro results, expressions levels of cIAP1, cIAP2 and XIAP were markedly downregulated in APG-1387-treated SKOV3 xenografts (Day-3 after administration, Fig. 8c). Similarly, apoptosis was induced by APG-1387 in vivo, as manifested by caspase-3 activation and PARP-1 cleavage (Fig. 8d).IHC assay further confirmed positive signals for TUNEL staining in APG-1387-treated tumors (Fig. 8e, f).

## Discussion

Apoptosis plays an essential role for organ development, homeostasis, and immune defense and provides mechanisms for the anti-cancer therapies. In recent years, apoptosis has been widely studied in relation to the treatment of malignant tumors. Apoptosis may also be inhibited by a variety of proteins including members of the inhibitors of apoptosis (IAP) family [24]. IAPs comprise a family of structurally similar proteins, such as cIAP1, cIAP2 and XIAP largely over expressed by most tumors. Restoring the apoptotic cell death machinery by pharmacological inhibition of IAPs proteins represents a compelling strategy for cancer therapy. APG-1387 is a novel Smac mimetic developed by Ascentage and currently being evaluated in phase I clinical trial. In our study, we investigated the in vitro and in vivo antitumor activity of APG-1387 in ovarian cancer. CCK-8 assay revealed that APG-1387 could significantly suppress the growth of ovarian cancer cells in a dose-dependent manner. Colony formation assay revealed that the number of cell clones decreased with increasing APG-1387 concentration, suggesting that the growth and viability of human ovarian cancer cells were largely inhibited by APG-1387. Our results showed that APG-1387 could effectively induce apoptosis in ovarian cancer. Previous studies have demonstrated that APG-1387 inhibits the growth and proliferation of NPC (nasopharyngeal carcinoma) cells in a dose-dependent manner [25]. Moreover, morphological changes in apoptotic characteristics, such as cellular shrinkage, rounding, poor adherence, and round floating shapes in harmine-treated cells were also observed by fluorescence microscopy. Apoptosis was also detected through fluorescein AnnexinV-FITC/PI double labeling [26]. Research indicates that APG-1387 induced cell apoptosis in a dose-dependent manner. DNA damage is an early event of apoptosis. Upon DNA damage, multiple events to promote cell survival and facilitate DNA repair [27]. If damage is excessive, programmed cell death to eliminate the afflicted cell [28]. APG-1387 treatment caused the formation of significant amounts of the phosphorylation of H_2_AX and increased apoptosis rates at concentration of 10 nM and 30 nM than the control group (*P* < 0.05). Two distinct apoptotic pathways have been identified: the intrinsic and extrinsic pathways [7]. The intrinsic and extrinsic pathways converge by activating the effector caspases-3/PARP, ultimately leading to the fragmentation of DNA with resultant cell death [29]. During apoptosis, caspase-3 is one of the key executioners of apoptosis in response to various stimuli [30] and play crucial roles in cell apoptosis [31]. It has been determined that a variety of chemotherapeutic agents induce apoptosis through the activation of caspase-3 and PARP [32, 33]. The activated-caspase-3 and cleaved-PARP causes morphological and biological apoptotic changes [34]. Our results revealed that APG-1387-induced apoptosis was related to down-regulation of IAPs expressions. The intrinsic and extrinsic pathways converge by activating the effector caspases-3/PARP. When APG-1387 was coadministered with or without Z-VAD-FMK, the growth inhibition of cell morphology and cell viability were partially blocked, and the activation of caspase-3 and PARP were also partially blocked. In previous studies, our team found that the in vitro antitumor effect of APG-1387 in NPC was RIPK1-dependent [25], caspase-8 is also involved in NF-κB signaling [35]. For example, homodimers formed by caspase-8 are associated with activation of NF-κB by RIPK1 [19]. Our study provided evidence that an RIP1-mediated apoptosis pathway could be activated in ovarian cancer cells. APG-1387 induced ovarian cancer cell death via decreasing expression of cIAP1, cIAP2, XIAP in a dose- and time-dependent manner. APG-1387 induced formation of a complex consisting of RIP1, FADD and caspase-8, resulting in apoptosis.

The recent trials of birinapant and DEBIO1143 (AT-406) reported patients showed a trend towards increased circulating TNF-α [15, 36]. Inaddition, the inhibition of NF-κB could sensitize the resistant to Smac mimetic/TNF-alpha treatment. TNF-alpha can increase the production of cytokines through binding to its receptors including TNF-alpha receptor 1 (TNFR1), which is ubiquitously expressed in human tissues and serves as a signaling receptor for TNF-alpha [37]. The impacts of NF-κB are pervasive as its effects extend across multiple systems. Research within the last few years has revealed that members of the NF-κB transcription factor regulate cell viability by activating genes involved in intrinsic pathways [38]. NF-κB1/p50 (p50, which is processed from p105), and NF-κB2/p52 (p52, which is processed from p100) make up the NF-κB family in mammals [39]. Mechanistic studies revealed that the secretion of TNF-alpha after ovarian cancer cells were treated with APG-1387 and found that the secretion of TNF-alpha was in time-dependent manner. Treatment with APG-1387 significantly decreased the expression of NF-κB p50, NF-κB p52 proteins as well as RIP1 which were required for NF-kB activation.

Autophagy is a catabolic pathway conserved among eukaryotic cells [40]. The conversion of LC3-I into LC3-II has been considered as a hallmark of autophagy [21]. Beclin1, as a crucial regulatory protein, regulates autophagosome membrane formation [41, 42]. ATG7 are required for the initiation of autophagy, and mediate phagophore expansion and autophagosome formation [43]. This process is initiated by formation of the phagophore, which expands and fuses to form a vehicle called an autophagosome. Autophagosomes eventually fuse with lysosomes, which degrade their contents. Therefore, autophagy allows cells to rapidly eliminate long-lived proteins and destructive organelles for energy recycling [44]. It’s primarily a process for cell protection, playing a pivotal role in cell survival, differentiation, development, and homeostasis [45]. In our study, we demonstrated that APG-1387 treatment increased the transition of LC3-I to LC3-II, increased the expression of Beclin1, decreased the expression of P62 and increased the accumulation of punctate LC3 in the cytoplasm in cells. Transfection of cells with Beclin1 siRNAs or ATG7 siRNAs blocked the accumulation of LC3-II after APG-1387 treatment, a result which indicating that APG-1387 induced the autophagy of ovarian cancer cells. In most cases, autophagy-associated death is accompanied with apoptosis [46]. Autophagy is considered as a double-edged sword with regard to genesis, development and the treatment of tumors as it kills tumor cells but also protect tumor cells against injury [18]. However, uncontrolled autophagy will gradually consume intracellular components and lead to cell death [47]. In recent years, many studies have reported that autophagy plays an important role in tumorigenesis and is becoming a key regulator of cancer survival [48, 49]. While the term of autophagic cell death is still a matter of dispute, accumulating evidence suggested the possibility of treating malignant tumors through autophagic regulation. Drugs that potentially modulate autophagy are increasingly being used in clinical trials, and screens are being performed for new drugs that can modulate autophagy for therapeutic purposes [21]. Cancer cells fight against external stimuli through autophagy, which is especially evident in solid tumor cells where blood supply is inadequate. Autophagy plays a role in promoting tumor cell growth and survival [50]. Autophagy also protects certain tumor cells from radiation damage [51]. It is speculated that the mechanism of this protective effect may be through autophagy to remove damaged macromolecules or mitochondria and other organelles, thereby protecting tumor cells from apoptotic apoptosis [52]. Our results suggest that APG-1387 induces autophagy while triggering apoptosis. APG-1387-induced autophagy plays a role in protecting cell survival and inhibition of autophagy potentiates cytotoxicity of APG-1387 in ovarian cancer cells.

In vivo, APG-1387 potently inhibited SKOV3 xenograft growth in nude mice, without causing apparent toxicities. Therefore, APG-1387 is an efficient anti-ovarian cancer agent. These observations indicate that APG-1387 may be effective as a single agent in patients with ovarian cancer.

## Conclusions

This research demonstrated that APG-1387 has a potent antitumor effect on ovarian cancer both in vitro and in vivo by inducing apoptosis. APG-1387 induced ovarian cancer cell death by modulating autophagy via regulating expression of IAP families. The preclinical activity and mechanism of APG-1387 in ovarian cancer indicated its potential as a novel therapeutic for the treatment of ovarian cancer.
