# Correction to: Novel smac mimetic APG-1387 elicits ovarian cancer cell killing through TNF-alpha, Ripoptosome and autophagy mediated cell death pathway

**DOI:** 10.1186/s13046-018-0774-7

**Published:** 2018-05-24

**Authors:** Bao-Xia Li, Heng-Bang Wang, Miao-Zhen Qiu, Qiu-Yun Luo, Han-Jie Yi, Xiang-Lei Yan, Wen-Tao Pan, Lu-Ping Yuan, Yu-Xin Zhang, Jian-Hua Xu, Lin Zhang, Da-Jun Yang

**Affiliations:** 1 State Key Laboratory of Oncology in South China, Collaborative Innovation Center for Cancer Medicine, Sun Yat-sen University Cancer Center, Guangzhou, 510060 China; 2 Department of Pharmacology, Fujian Provincial Key Laboratory of Natural Medicine Pharmacology, School of Pharmacy, Fujian Medical University, Fuzhou, 350108 China; 3 Ascentage Pharma Group Corp., Ltd, Taizhou, 225309 China; 4 Department of Medical Oncology, State Key Laboratory of Oncology in South China, Collaborative Innovation Center for Cancer Medicine, Sun Yat-Sen University Cancer Center, Guangzhou, 510060 China; 5 Departments of Clinical Laboratory, State Key Laboratory of Oncology in South China, Collaborative Innovation Center for Cancer Medicine, Sun Yat-Sen University Cancer Center, 651 Dongfeng Road East, Guangzhou, 510060 China

## Correction

In the publication of this article [1], there was an error in the Methods, Cell cultures and reagents section: the Methods, Cell cultures and reagents section: ‘The following primary antibodies were used: P62(#8025), phospho-H2AX(γ-H2AX;#9718), caspase-8(#9746), RIP1(#3493s), Beclin1(3738s), ATG7 (#2631S), PARP (#9546S), caspase-3(#9665s), cIAP1 (#7065s), cIAP2(3130s), XIAP(#14334), FADD(#2782S), phospho-NF-κBp105/p50(4806S), NF-κB2p100/p52(#4882S) and TNFR1(#3736S) were purchased from Cell Signaling Technology Inc.; GAPDH (#sc-47724) from Santa Cruz Biotechnology (SC); LC3 (#NB100–2220) from Novus Biologicals’.

Should instead read: ‘The following primary antibodies: P62 (#8025), phospho-H2AX (γ-H2AX;#9718), caspase-8 (#9746), RIP1 (#3493s), Beclin1 (3738s), ATG7 (#2631S), PARP (#9546S), caspase-3 (#9665s), cIAP1 (#7065), cIAP2 (3130s), XIAP (#14334), FADD (#2782S), NF-κBp105/p50 (4806S), NF-κB2p100/p52 (#4882S), TNF-α (#6945s), TNF-α neutralizing antibody (7321s), and TNFR1 (#3736S) were purchased from Cell Signaling Technology Inc.; GAPDH (#sc-47724) was from Santa Cruz Biotechnology (SC); LC3 (#NB100–2220) was from Novus Biologicals. Z-VAD-FMK (#V116) and Necrostatin-1(#N9037) were from Sigma. IKK-16(#S2882) from Selleck’.

In the Results, APG-1387 is RIP1-dependent in ovarian cancer induced apoptosis section there was an error: ‘We examined the protein levels of caspase-8/RIP1 by western blot, as shown in Fig. 4a’.

Should instead read: ‘We examined the protein levels of caspase-8/RIP1 by western blot. APG-1387 triggered the activation of caspase-8 and downregulated the protein level of RIP1, as shown in Fig. 4a’.

In the Results, APG-1387 induces apoptotic cell death through engagement of TNFR1 by TNF-alpha signaling pathway section there was an error: ‘We have investigated the expression of NF-κB1/p50 and NF-κB2/p52 by western blot after cells were incubated with various concentrations of APG-1387’.

Should instead read: ‘These results demonstrate that TNFα signaling is required for APG-1387-induced apoptotic cell death. Next, we have investigated the expression of NF-κB1/p50 and NF-κB2/p52 by western blot after cells were incubated with various concentrations of APG-1387’.

A section header contained an error: ‘APG-1387-induced autophagy’

Should instead read: ‘APG-1387 induces autophagy in ovarian cancer’

A section header contained an error: ‘APG-1387-induced apoptosisby modulating autophagy’

Should instead read: ‘Inhibition of autophagy sensitizes ovarian cancer cells to A PG-1387-induced apoptosis’.

In the Discussion section there was an error: ‘APG-1387 is a novel Smac mimetic. In our study, we investigated the molecular mechanisms underlying the inhibitory effect on the growth of ovarian cancer cell lines treated with varying concentrations of APG-1387’.

Should instead read: ‘Restoring the apoptotic cell death machinery by pharmacological inhibition of IAPs proteins represents a compelling strategy for cancer therapy. APG-1387 is a novel Smac mimetic developed by Ascentage and currently being evaluated in phase I clinical trial. In our study, we investigated the in vitro and in vivo antitumor activity of APG-1387 in ovarian cancer’.

In the Discussion section there was an error: ‘Our results suggest that APG-1387 induces autophagy during apoptosis. Autophagy plays a role in protecting cell survival. We have also found that it was effective as a single agent in vivo models. Treatment with APG-1387 induced potent cytotoxic and anti-proliferative activity against established and human ovarian cancer cells’.

Should instead read: ‘Our results suggest that APG-1387 induces autophagy while triggering apoptosis. APG-1387-induced autophagy plays a role in protecting cell survival and inhibition of autophagy potentiates cytotoxicity of APG-1387 in ovarian cancer cells’.

Figure 7 caption had an an error: ‘APG-1387-induced autophagy promote cell death in SKOV3 cells’.

Should instead read: ‘Fig. 7 APG-1387-induced autophagy promotes cell survival in SKOV3 cells’.

In the Funding section there was an error due to the fact some additional funds were missing.

Should instead additionally read: ‘the Project of International Science and Technology Cooperation of Fujian Province (Grant number. 2015I0002); and the Joint Funds for the innovation of science and Technology, Fujian province (Grant number. 2016Y91030065). The correct Figs. 2, 3 and 6 are presented here:

**Fig. 2:**
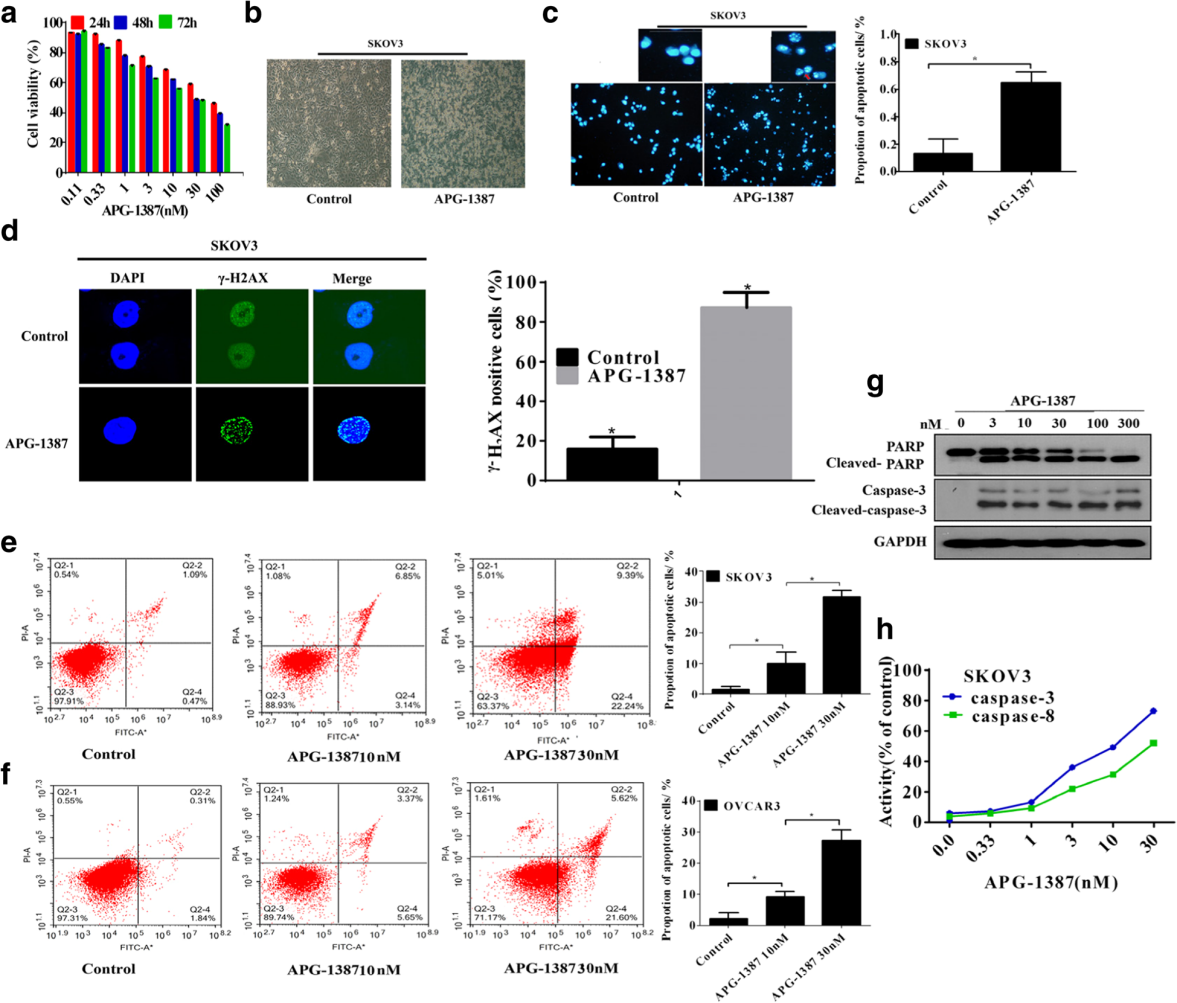
Effects of APG-1387 on apoptosis in ovarian cancer. **a** APG-1387 inhibited the proliferation of SKOV3 cell line. They were treated with the indicated concentrations of APG-1387 for 24, 48, 72 h. Cell viability was determined by the CCK-8 assay. **b** Morphology of SKOV3 cells exposed to APG-1387(0, 10 nM) photographed under a fluorescence microscope (original magnification× 10). **c** APG-1387-induced apoptosis in SKOV3 cells was assessed by Hoechst33258 staining. Morphology of SKOV3 cells exposed to APG-1387 at different concentrations photographed under a fluorescence microscope (original magnification × 10). Condensated and fragmented nuclears were the mean ± SEM of 5 randomized areas. *P* < 0.01. **d** SKOV3 cells were treated with 10 nM APG-1387 for the indicated times. The cells were stained for phosphorylated H_2_AX and DAPI, then were analyzed by fluorescence microscopy (original magnification × 200). γ-H_2_AX positive spots were the mean ± SEM of 5 randomized areas. *P* < 0.01. **e**, **f** SKOV3 and OVCAR3 cells were exposed to various concentrations of APG-1387 (0, 10, 30 nM) for 24 h followed cell apoptosis analysis by flow cytometry. **g** Western blot analysis of caspase-3/PARP SKOV3 cells were treated withAPG-1387 (0, 3, 10, 30, 100, 300 nM) for 24 h. The data shown are representative of three different experiments. **h** SKOV3 cells were stimulated with APG-1387 for indicated periods of concentrations, caspase activation were tested by caspase activity assay

**Fig. 3:**
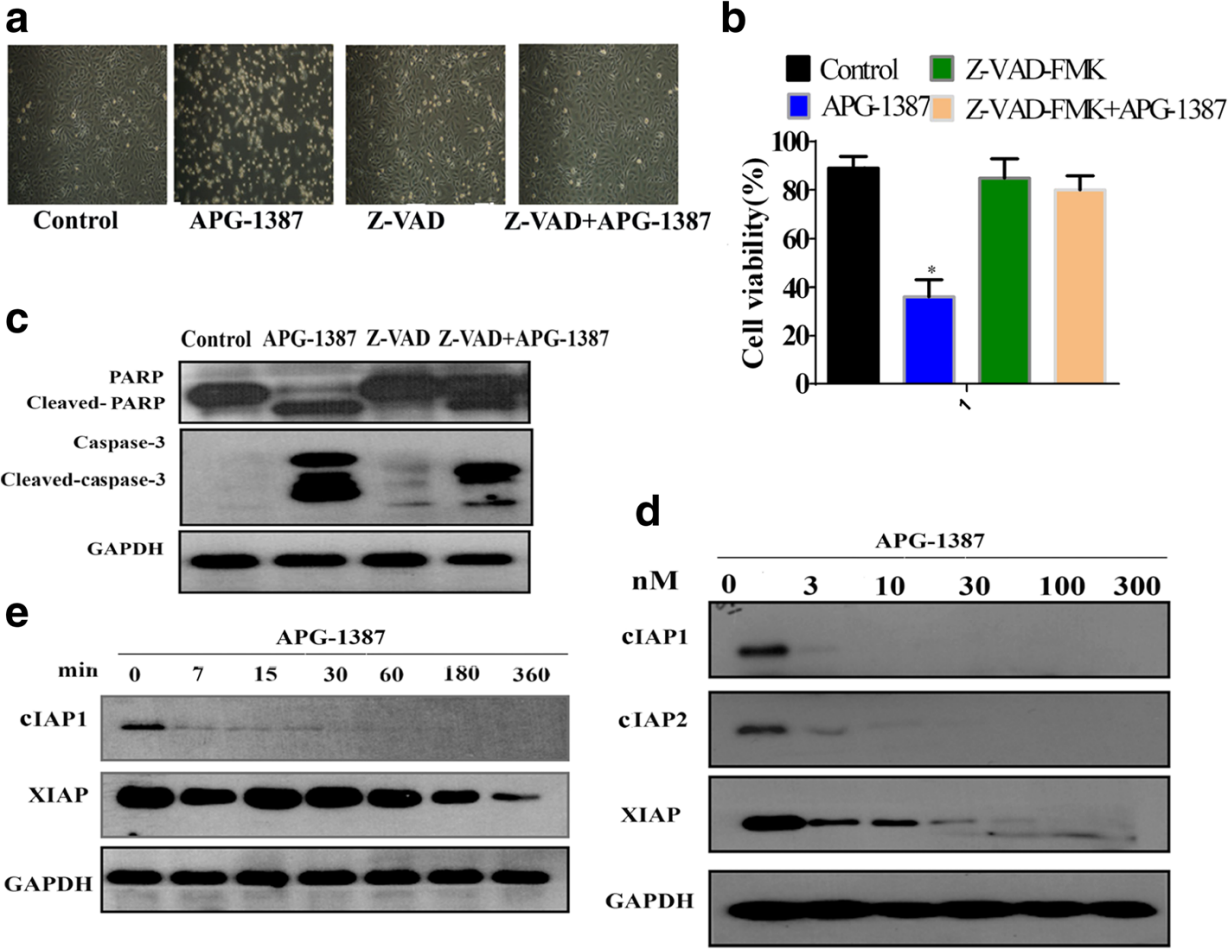
APG-1387-induced apoptosis in caspase dependent manner. **a** Cells with or without addition of Z-VAD-FMK. Morphology of cells exposed to different treatment groups photographed under a fluorescence microscope (original magnification × 10). **b** APG-1387 was coadministered with or without addition of caspase inhibitor (Z-VAD-FMK). Cell viability was determined by the CCK-8 assay. **c** Western blot analysis of the effect of APG-1387 with or without addition of Z-VAD-FMK on caspase-3/PARP expression level in SKOV3 cells. **d** Western blot analysis of the expression levels of IAPs at different concentrations of APG-1387 in SKOV3 cells. **e** Cells were treated with different time points, and the effect of APG-1387 on IAP family members expression level was determined by western blot. Data represent one of three experiments yielding similar results

**Fig. 6:**
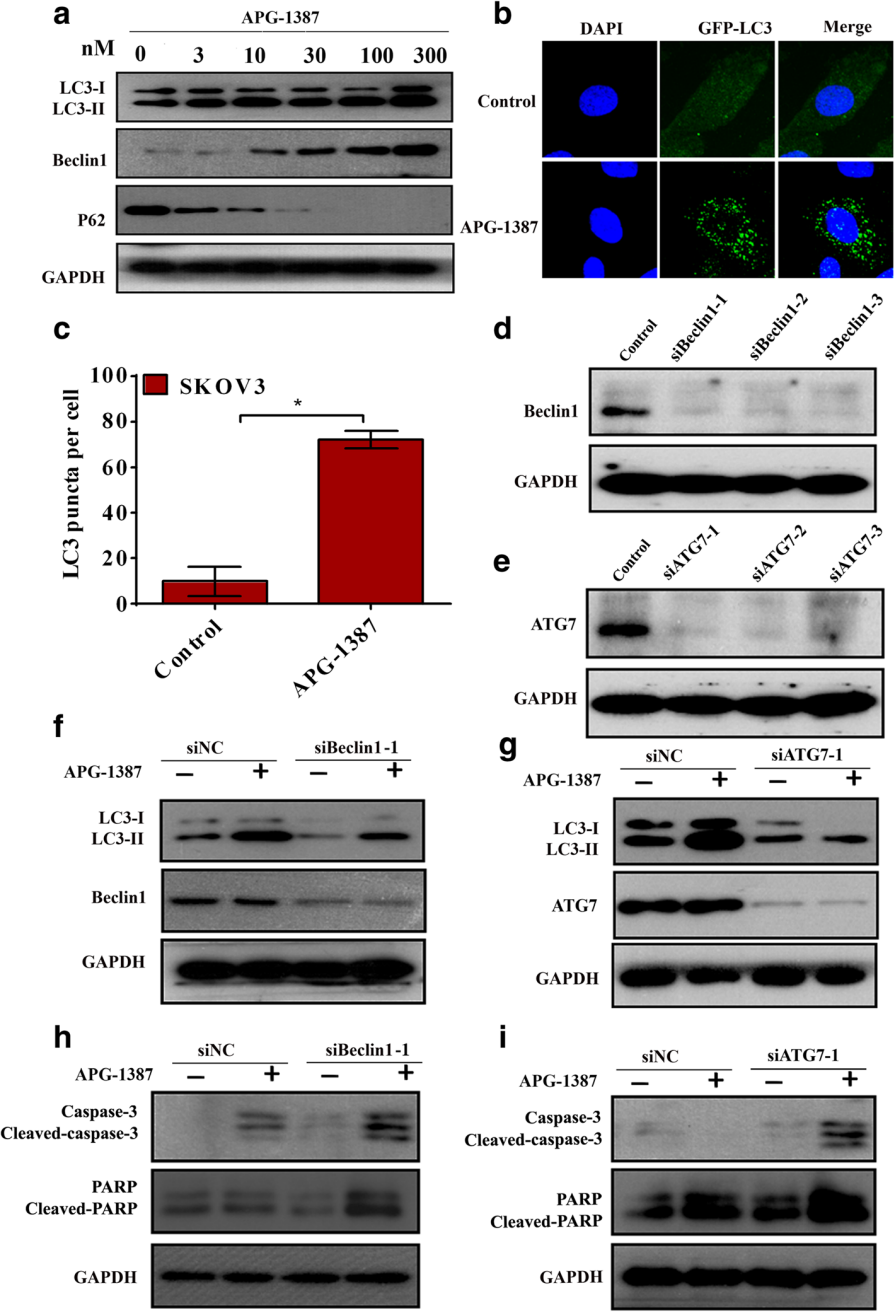
APG-1387 induces autophagy in ovarian cancer cells. **a** The expression of LC3, Beclin1 and P62 was measured by western blot. Cells were treated with APG-1387(0, 3, 10, 30, 100, 300 nM) for 24 h. **b** Cells were transfected with GFP-LC3 plasmids, and then maintained in media with or without 3 nM APG-1387 for 24 h. The cells were then stained with DAPI and analyzed by fluorescence microscopy. **c** Statistical analysis of the percentage of LC3 puncta per cell. Columns, mean (*n* = 3); bars, SD. *P < 0.01 vs. untreated group. LC3 puncta per cell were quantified. **d** Cells were transfected with Beclin1 siRNAs. Western blot was used to detect the expression of Beclin1. **e** Cells were transfected with ATG7 siRNAs. Western blot was used to detect the expression of ATG7. **f** Cells were transfected with Beclin1 siRNAs. After 24 h treatment with or without 3 nM APG-1387, western blot analysis was performed for indicated proteins. **g** Cells were transfected with ATG7 siRNAs. After 24 h treatment with or without 3 nM APG-1387, western blot analysis was performed for indicated proteins. **h** Western blot analysis was performed for indicated proteins in cells transfected with siBeclin-1 and treated with 10 nM APG-1387. **i** Western blot analysis was performed for indicated proteins in cells transfected with siATG7–1 and treated with 10 nM APG-1387

This has now been updated in the original article [1].
